# Genome-wide DNA methylation profiling shows a distinct epigenetic signature associated with lung macrophages in cystic fibrosis

**DOI:** 10.1186/s13148-018-0580-2

**Published:** 2018-12-10

**Authors:** Youdinghuan Chen, David A. Armstrong, Lucas A. Salas, Haley F. Hazlett, Amanda B. Nymon, John A. Dessaint, Daniel S. Aridgides, Diane L. Mellinger, Xiaoying Liu, Brock C. Christensen, Alix Ashare

**Affiliations:** 1 Department of Epidemiology, Geisel School of Medicine at Dartmouth, Lebanon, NH USA; 2 Department of Molecular and Systems Biology, Geisel School of Medicine at Dartmouth, Lebanon, NH USA; 3 Department of Medicine, Dartmouth-Hitchcock Medical Center, Lebanon, NH USA; 4 Program in Experimental and Molecular Medicine, Geisel School of Medicine at Dartmouth, Hanover, NH USA; 5 Department of Microbiology and Immunology, Geisel School of Medicine at Dartmouth, Dartmouth-Hitchcock Medical Center, Lebanon, NH USA; 6 Department of Pathology and Laboratory Medicine, Dartmouth-Hitchcock Medical Center, Lebanon, NH USA; 7 Department of Community and Family Medicine, Geisel School of Medicine at Dartmouth, Lebanon, NH USA

**Keywords:** Lung macrophages, Bronchoalveolar lavage, DNA methylation, Epigenetics

## Abstract

**Background:**

Lung macrophages are major participants in the pulmonary innate immune response. In the cystic fibrosis (CF) lung, the inability of lung macrophages to successfully regulate the exaggerated inflammatory response suggests dysfunctional innate immune cell function. In this study, we aim to gain insight into innate immune cell dysfunction in CF by investigating alterations in DNA methylation in bronchoalveolar lavage (BAL) cells, composed primarily of lung macrophages of CF subjects compared with healthy controls. All analyses were performed using primary alveolar macrophages from human subjects collected via bronchoalveolar lavage. Epigenome-wide DNA methylation was examined via Illumina MethylationEPIC (850 K) array. Targeted next-generation bisulfite sequencing was used to validate selected differentially methylated CpGs. Methylation-based sample classification was performed using the recursively partitioned mixture model (RPMM) and was tested against sample case-control status. Differentially methylated loci were identified by fitting linear models with adjustment of age, sex, estimated cell type proportions, and repeat measurement.

**Results:**

RPMM class membership was significantly associated with the CF disease status (*P* = 0.026). One hundred nine CpG loci were differentially methylated in CF BAL cells (all FDR ≤ 0.1). The majority of differentially methylated loci in CF were hypo-methylated and found within non-promoter CpG islands as well as in putative enhancer regions and DNase hyper-sensitive regions.

**Conclusions:**

These results support a hypothesis that epigenetic changes, specifically DNA methylation at a multitude of gene loci in lung macrophages, may participate, at least in part, in driving dysfunctional innate immune cells in the CF lung.

**Electronic supplementary material:**

The online version of this article (10.1186/s13148-018-0580-2) contains supplementary material, which is available to authorized users.

## Background

The role of the innate immune system in the pathogenesis of cystic fibrosis (CF) lung disease has been an emerging research focus [1–3]. The inability to regulate both the chronic infections and an excessive inflammatory response suggests that the innate immune system is dysfunctional in CF [1]. Studies have revealed numerous physiologic defects associated with CF macrophages including dysregulation of phagocytic/signaling receptors [4], hyper-responsiveness to microbial stimuli [5–7], and impairment in removal of apoptotic cells [8, 9]. Additionally, increased levels of inflammatory mediators, typically secreted from macrophages, in sputum and bronchoalveolar lavage (BAL) fluid have been described in CF patients [10–12].

The multitude of biological processes seemingly affected in CF macrophages begs the following question: is there a broader epigenetic mechanism influencing a myriad of biological functions in CF macrophages? Epigenetics is the study of heritable changes in gene function caused by mechanisms other than changes in the underlying DNA sequence [13]. The most widely studied of the epigenetic modifications is DNA methylation [14]. Epigenetic mechanisms have emerged as modulators of host defenses that can lead to a more prominent immune response and shape the course of inflammation in the host, both driving the production of specific inflammatory mediators and controlling the magnitude of the host response [15].

To examine possible underlying epigenetic mechanisms related to dysregulation of innate immunity in CF, we initiated an epigenome-wide DNA methylation profiling study from primarily lung macrophages isolated from BAL fluid in subjects with and without CF.

## Results

### Subject characteristics

BAL samples were sequentially taken from the right upper lobe (RUL) and right lower lobe (RLL) of heathy (*n* = 4) and CF subjects (*n* = 4). Subject characteristics are listed in Table 1.

**Table 1:** Subject characteristics

Patient number	Status	Age	Sex	Genotype	FEV_1_ (%)	Microbiology	Antibiotic and/or modulator therapy
1	CF	18	Male	F508del/Y1092X	77	*S. aureus* many mixed	None
2	CF	24	Male	F508del/F508del	96	*S. aureus*	Aztreonam, colistimethate, doxycycline, ivacaftor/lumacaftor
3	CF	28	Female	F508del/F508del	91	*A. xylosoxidans* moderate mixed	Colistimethate, tobramycin
4	CF	18	Female	F508del/F508del	88	*S. aureus* many mixed *A. xylosoxidans*, moderate mixed	Colistimethate, tobramycin, ivacaftor/lumacaftor
5	Healthy	26	Female				
6	Healthy	33	Male				
7	Healthy	25	Female				
8	Healthy	20	Female				

*S. aureus Staphylococcus aureus*, *A. xylosoxidans Achromobacter xylosoxidans*

Gender and age distribution were as follows: two male and two female CF subjects with a mean age of 22.0 + 4.90 years; healthy subjects (three females/one male) had a mean age of 26.0 + 5.35 years. Three CF individuals were genotype *F508del/F508del*, and one participant had genotype *F508del/Y1092X*. Forced expiration volume (FEV_1_) measurement range was 77–96% across the CF study group. The lung microbiology based on standard BAL culture was recorded for each CF patient. Three subjects cultured positive for *Staphylococcus aureus*, and two cultured positive for *Achromobacter xylosoxidans*. Three CF subjects were on antibiotic and/or modulator therapy including one or more of the following: inhaled aztreonam, inhaled colistimethate, oral doxycycline, inhaled tobramycin, or ivacaftor/lumacaftor.

### DNA methylation landscape between CF and healthy individuals

**Fig. 1:**
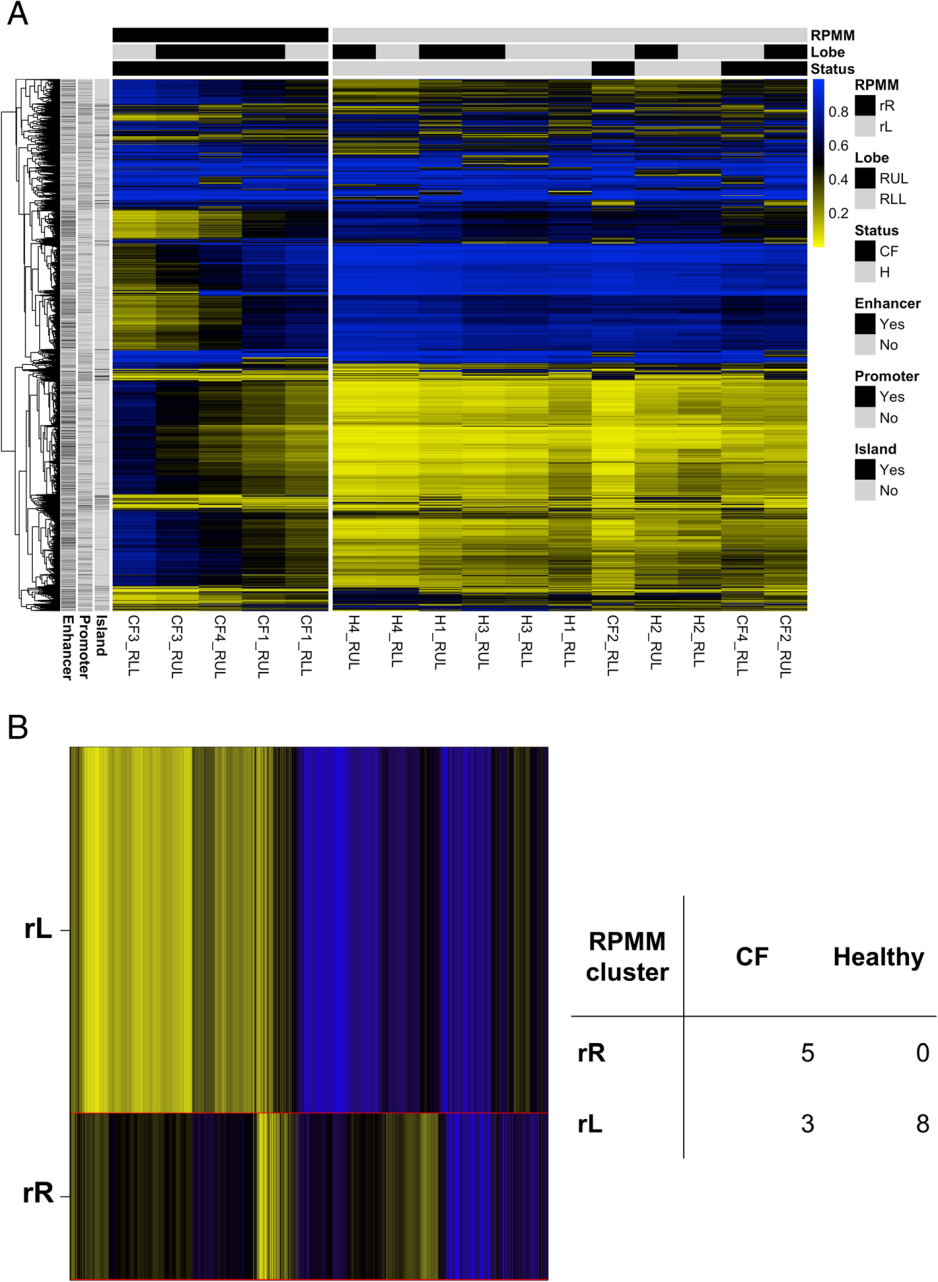
DNA methylation landscape in CF versus healthy controls. Recursively partitioned mixture model (RPMM) of the 10,000 CpGs (rows) with greatest sample variance across subjects. Individual samples 1–16 are shown in columns with sample status bar at the top: black (CF) and gray (healthy). Blue color represents increased sample methylation (**a**). RPMM determined the similarity of methylation among subjects resulting in two methylation classes L and R (**b**). Methylation class membership was associated with class status; inset contingency table depicts subject distribution in each class via a two-tailed Fisher’s exact test (*P* = 0.026)

We determined methylation subclasses with an unsupervised, model-based method, recursively partitioned mixture model (RPMM, Fig. 1). RPMM clustering of the 10,000 CpG sites with the highest variance in DNA methylation revealed greater adjacency of CF samples, as well as the clustering of healthy samples. In addition, CF subjects which showed pronounced heterogeneity (Fig. 1a). Subsequently, we formally tested BAL sample disease status against DNA methylation cluster membership. All BAL samples predicted to be in RPMM cluster *L* were from healthy subjects, and the majority of BAL samples in RPMM cluster *R* were from CF subjects (Fig. 1b, *P* = 0.026, two-tailed Fisher’s exact test).

### Cellular composition and heterogeneity profiling

**Fig. 2:**
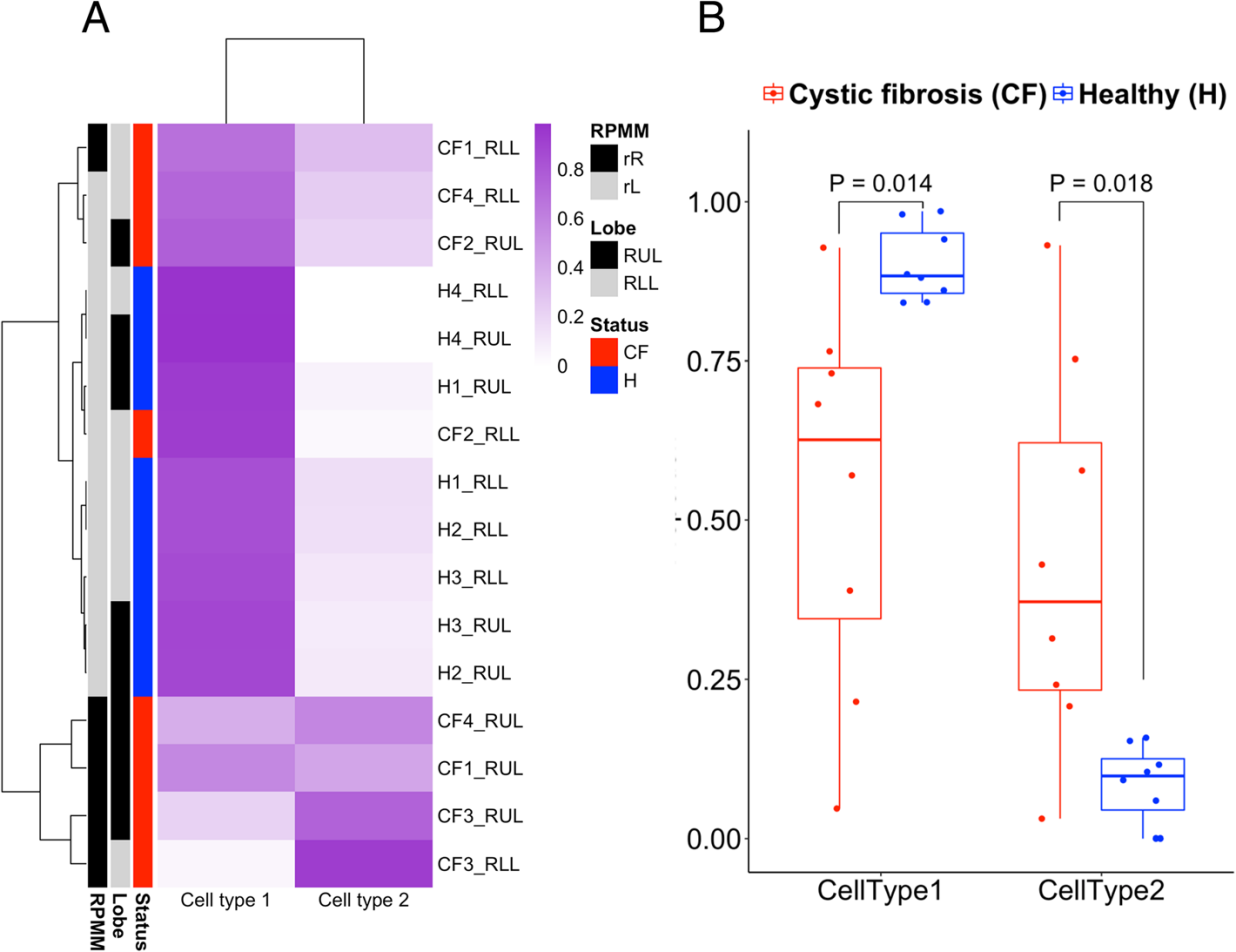
Cellular composition and heterogeneity profiling. Heat map illustrates reference-free deconvolution (RefFreeEWAS) of putative cell type and size proportions across the *n* = 16 samples included in this study (**a**). Healthy subjects had higher proportions of putative cell type/size 1 (*P* = 0.014), and CF subjects had a higher proportion of cell type/size 2 (*P* = 0.018) (**b**)

**Fig. 3:**
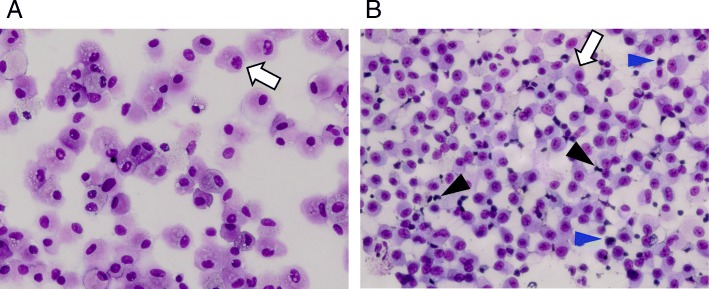
Bronchoalveolar lavage cell cytospins. Bronchoalveolar lavage (BAL) samples were obtained from tertiary airways in the right upper lobe of subjects. BAL cells were isolated and prepared as cytospins as described in the “Methods” section. BAL cells from healthy subjects are a mostly homogeneous population of cells (lung macrophages (LM)) with oval to reniform nuclei and abundant cytosol (open arrow) (**a**). Cytospins of BAL cells from CF subjects (CD15-depleted) (**b**) show a majority population of LMs (open arrow) as well as smaller roundish cells with darker staining nuclei and less cytosol (blue arrowheads) and cells containing variably shaped and stained nuclei (black arrowheads). Images shown are representative of multiple subjects

To assess the potential contribution of BAL sample cell type heterogeneity on DNA methylation, we utilized an approach to cell type deconvolution that does not require a reference library of differentially methylated loci for specific cell types [16]. Across all samples, the reference-free cell type deconvolution identified two putative cell types (Fig. 2a). Putative cell type 1 proportions were higher in healthy subjects compared to CF subjects (Fig. 2b, *P* < 0.05), and putative cell type 2 proportions were lower in healthy controls compared to CF subjects (*P* < 0.05). To confirm cell-type heterogeneity in CF subjects, cytospins were performed on RUL BAL cells isolated from a second group of healthy subjects and a second group of CF subjects (*n* = 3) genotypes (*E60X/A455E*, *R1162X/W1282G*, and *F508del/F508del*). BAL cells from healthy subjects have characteristic lung macrophage “fried egg” or monocytoid appearance with reniform nuclei and ample cytosol (Fig. 3a, open arrow), with this cell phenotype comprising > 95% of the total cell population. The majority of BAL cells from CF subjects were similar to the macrophages seen in healthy subjects. However, the CF subjects had subpopulations of macrophages that were phenotypically diverse in size and appearance (Fig. 3b), as previously demonstrated in other respiratory disease states [17].

### Epigenome-wide association analysis reveals differentially methylated loci

**Fig. 4:**
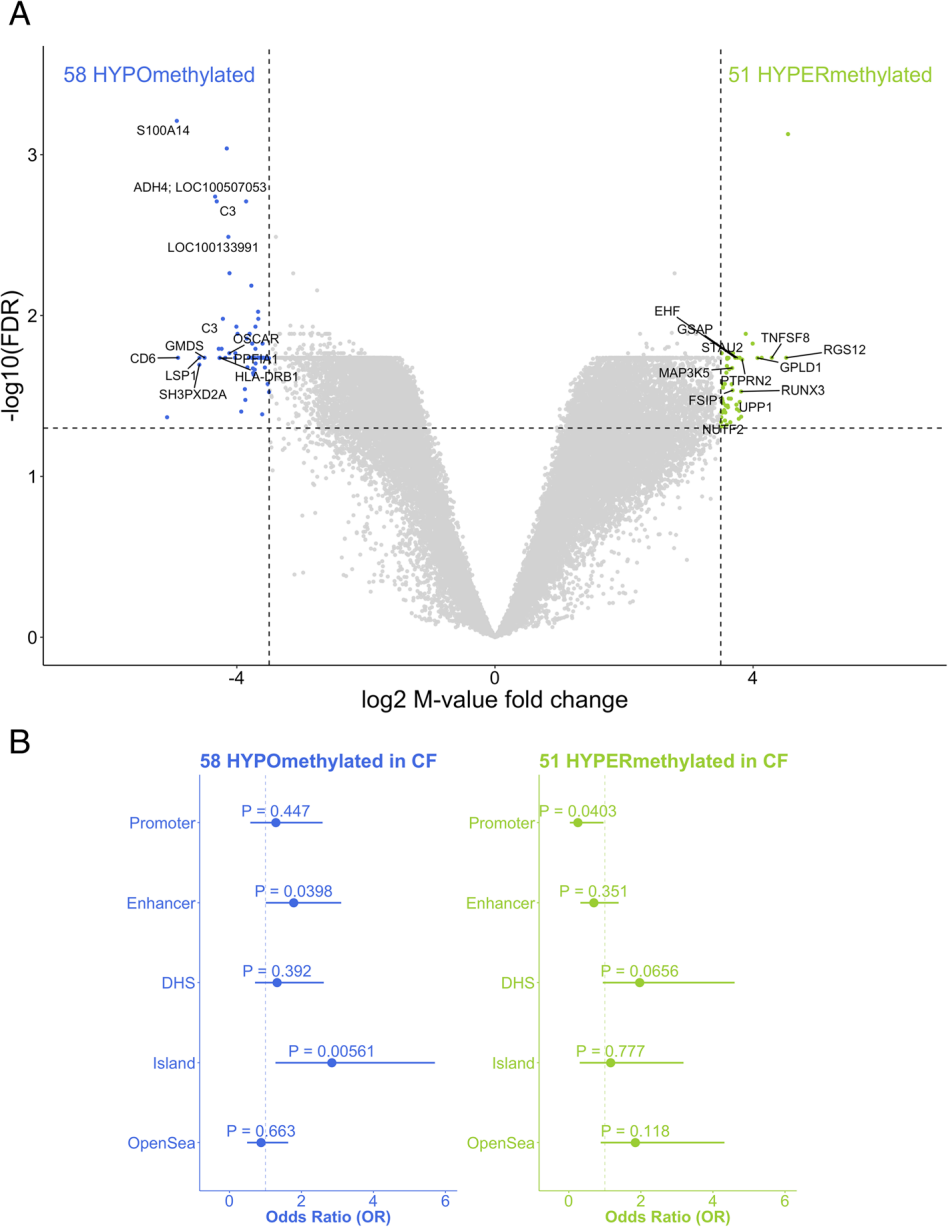
Epigenome-wide differential methylation in CF. Comparative analysis of DNA methylation in CF and healthy subjects identified 109 differentially methylated CpGs (FDR *P* < 0.1, **a**). CpG hyper-methylated in CF compared to controls (green) and hypo-methylated CpGs (blue) are plotted as log_2_ fold increase or decrease in methylation *M* value (*x*-axis) versus log_10_ FDR-adjusted *P* value (*y*-axis). Statistically significant CpGs associated with specific genes are labeled, and unlabeled points represent CpGs associated with no known gene at that location. Enrichment of differentially methylated CpGs to genomic and transcriptional context is shown in forest plots (**b**), illustrating that hypo-methylated CpGs in CF BAL are enriched for enhancer regions and CpG islands and that hyper-methylated CpGs in CF BAL are under-represented for gene promoter regions

Next, we investigated the relationship between DNA methylation and CF disease status in BAL samples. Because we identified differential proportions of putative cell types in CF subjects compared with controls, to identify differential methylation independent of cell type, we fit linear mixed effects models comparing methylation of the 26,733 most variable CpG sites with CF disease status, adjusted for subject age and sex, and included a term to account for repeat measurements from the same subject (RUL, RLL). We identified 109 differentially methylated CpGs, of which 51 are hyper-methylated and 58 are hypo-methylated in CF cases compared with controls (FDR-adjusted *P* < 0.1, |log_2_FC_*M* value_| ≥ 3.50, Fig. 4a). There is a 31.6% median increase and 27.2% median reduction in the proportion of methylated alleles (beta-values) for differentially hyper- and hypo-methylated loci, respectively. The top five hypo- and hyper-methylated CpGs associated with known genes are shown in Additional file 1: Table S1.

We utilized targeted next-generation bisulfite sequencing (tNGS) for the validation of the EPIC methylation array. Genes that showed some of the greatest Δ-beta values, including *CD6*, *HOOK2*, *LSP1*, *RGS12*, *SH3PXD2A*, and *UPP1*, were selected to compare CpG methylation in CF vs. healthy subjects. Δ-Beta methylation values were consistent between the array and the sequencing approaches to measure DNA methylation (Table 2). Additionally, an example of tNGS assay design is shown for *LSP1* in Additional file 2: Figure S1, and detailed information about the tNGS assays performed including chromosomal location, number of CpG sites, average sample reads, and Δ-beta values across the entire assay is shown in Additional file 3: Table S2.

**Table 2:** Comparison of % methylation changes in CF subjects at gene-specific CpGs: EPIC Δ-beta vs targeted NGS Δ-beta value

Gene	Location	EPIC Δ-beta value	tNGS Δ-beta value
CD6	cg26427109	(−) 0.42	(−) 0.344
HOOK2	cg11738485	(−) 0.32	(−) 0.32
LSP1	cg18723409	(−) 0.44	(−) 0.292
RGS12	cg03132824	(+) 0.40	(+) 0.291
SH3PXD2A	cg06888746	(−) 0.28	(−) 0.184
UPP1	cg10317717	(+) 0.24	(+) 0.263

Next, we investigated the distribution of differentially methylated loci in CF subjects versus controls by genomic and transcriptional context. Among the hypo-methylated CpGs, enhancer regions and CpG islands were enriched (all OR > 1 and *P* < 0.05), and promoter-associated loci were significantly depleted in the hyper-methylated loci (OR = 0.25, *P* < 0.05) (Fig. 4b and Additional file 4: Table S3).

Unique genes associated with the top 5% (1337/26,377) most significant hyper-methylated and hypo-methylated CpGs were used as separate inputs for Kyoto Encyclopedia of Genes and Genomes (KEGG) pathway analysis against the 8515-gene universe associated with the 26,733 background loci (Additional file 5: Table S4).

In addition, we noted that 34.8% (38/109) differentially methylated loci do not track to any known genes. Compared to all CpGs tested for differential methylation, these “gene-less” CpG sites were over twice as likely as to co-occur with known single nucleotide polymorphisms (SNPs) (OR = 2.60, 95% CI = 1.27–5.18, *P* = 5.09E−3, Fisher’s exact test). We have provided the complete list of DMPs with FDR < 0.05 as Additional file 6: Table S5.

## Discussion

The primary objective of this study was to examine DNA methylation changes in CF BAL cells, primarily lung macrophages to gain mechanistic insight into innate immune regulation in cystic fibrosis. Our study is the first, which we are aware of, to report DNA methylation differences from lung macrophages isolated from CF subjects compared to healthy controls. One study has previously shown altered DNA methylation at a select number of lung modifier genes in nasal epithelial cells and whole blood of CF subjects [18]. Other studies have focused only on either histone-based macrophage epigenome profiling associated with macrophage phenotype [19–21] or the mapping of the lung proteome in cystic fibrosis [22]. However, no investigation to date has used epigenome-wide DNA methylation profiling to study cystic fibrosis BAL cells. Our cell collection methodology (flexible bronchoscopy) is a rare approach used in CF research studies and affords us a unique opportunity to analyze innate immune cells isolated directly from the airway and alveolar space.

Our initial strategy was to collect BAL cells of both CF and healthy subjects to analyze epigenome-wide DNA methylation patterns. DNA methylation profiling revealed a distinct clustering pattern associated with CF subjects as compared to healthy subjects. Additionally, we identified 109 differentially methylated CpGs, of which 51 are hyper-methylated and 58 are hypo-methylated. Hyper-methylated CpGs included those associated with genes such as *TNFSF8* and *RUNX3*. *TNFSF8*, also known as *CD30L*, has been suggested to contribute to pro-inflammatory immune response in a cross-talk role between innate and adaptive immune cells [23] and has noted to be expressed at high levels in alveolar macrophages from sarcoid subjects [24]. The transcription factor RUNX3 has been shown to regulate chemokines CCL5, CCL19, and CXCL11, chemotactic molecules with the potential to recruit various leukocytes into inflammatory sites [25, 26]. Hypo-methylated CpGs in CF patients compared to controls included those associated with genes such as *S100A14*, *LSP1*, and *OSCAR*. S100A14 is a member of a S100 family of proteins, a family of calcium-binding cytosolic proteins composed of 25 known members that have a broad range of intracellular and extracellular functions including regulating calcium balance, cell apoptosis, migration, and proliferation [27, 28]. Studies demonstrate that when released into extracellular space, S100 proteins have crucial activities in the regulation of immune homeostasis, post-traumatic injury, and inflammation [28]. Another hypo-methylated CpG is associated with the gene for leukocyte-specific protein 1 (*LSP1*). LSP1 is an actin-associated protein expressed in macrophages, neutrophils, and endothelial cells and has been localized to nascent phagocytic cups during Fcγ receptor-mediated phagocytosis, where it displays the same spatial and temporal distribution as actin filaments. Downregulation of LSP1 severely reduces phagocytic activity of macrophages, clearly indicating a crucial role for this protein in Fcγ receptor-mediated phagocytosis [29]. OSCAR, an immuno-receptor for surfactant protein D (SP-D), has been found in alveolar macrophages and together with SP-D contributes to lung homeostasis and innate mucosal defense [30]. In humans, OSCAR has been reported to be expressed on monocytes, macrophages, neutrophils, and dendritic cells [31] and shown to enhance the pro-inflammatory response of monocytes [31, 32].

Kyoto Encyclopedia of Genes and Genomes (KEGG) analysis of the top 10 pathways for 803 unique genes associated with the top 5% CpGs based on *P* value revealed pathways such as biosynthesis of unsaturated fatty acids, glycerolipid metabolism, Fc gamma R-mediated phagocytosis, and Fc epsilon RI signaling as the top CpG-gene-associated pathways likely affected in CF subjects based on changes in DNA methylation pattern.

We were able to identify enrichment of hyper- or hypo-methylated loci to specific genomic contexts suggesting their importance for gene regulation. Hypo-methylated CpGs in CF subjects were enriched for putative enhancer regions and in CpG islands, regions of high frequency of CpG sites [33]. Interestingly, enhancer regions are classically defined as *cis*-acting DNA sequences that can increase the transcription of genes. They generally function independently of orientation and at various distances from their target promoter (or promoters). Furthermore, they do not necessarily act on the respective closest promoter but can bypass neighboring genes to regulate genes located more distantly along a chromosome [34]. In some cases, individual enhancers have been found to regulate multiple genes [35].

Myeloid regulatory cells (MRC) have come into focus recently in lung disease including cystic fibrosis [36, 37] and in bacterial infections [38, 39]. Our observation of multiple cell sizes/types (CD15 (−)) in our CF population suggests that subpopulations of macrophages or even MRCs could be contributing to the observed differences in putative cell types in BAL between CF subjects and controls. To control for this, we performed a reference-free deconvolution of putative cell type proportions from the EPIC DNA methylation data set. Semi-supervised reference-free methods allow estimating proportions of cell types in the absence of a known or reliable reference of purified cell types [40]. Specifically, RefFreeEWAS first regresses out the effect of the phenotype of interest on the data and then uses a singular value decomposition on an augmented matrix based on the estimated regression and residual variation matrix [16]. Unlike other reference-free methods, RefFreeEWAS does not assume that the top components of data variation are associated with cell-type composition. Instead, it assumes that the top components in the regression and residual variation space are the cell types present in the bio-specimen. While it is possible that the putative cell types identified using reference-free deconvolution may represent distinct terminally differentiated cell types, it is also possible that cells with different activation states or in different stages of differentiation are captured by one or more putative cell types. With the apparent identification of more than one cell subtype in the DNA methylation data, we conducted follow-up studies on additional CF subjects for confirmation. We performed cytospins to visualize BAL cells before and after CD15 negative selection on CF and healthy subject BAL fluid and noticed a heterogeneous cell population isolated from CF subjects. This observation is consistent with our previous work suggesting a size range of CD206(+) CF lung macrophages as identified by forward scatter height using flow cytometry [41]. Additionally, a recent study in COPD identifies “small” and “large” cells from alveolar spaces and lung interstitium in lung resection tissue of COPD subjects [17]. Although our study suggests the presence of these macrophage subpopulations in the CF lung, it is beyond the scope of this work to specifically identify these subpopulations and will be the focus of future studies.

Although our study has a limited sample size, this is the first study to report differential DNA methylation associated with cystic fibrosis using a whole-genome approach. In CF BAL cells, we identified a substantial number of differentially methylated CpGs after adjusting for potential confounders. Further, the extent of observed differential methylation was quite high and highlights their biological relevance. Taken together, the differential methylation status of these genes might indicate differential gene expression and imply a unique biology of the CF lung microenvironment.

## Conclusions

Through the use of epigenome-wide DNA methylation profiling, we have identified 109 differentially methylated CpG loci in CF BAL cells. In addition, unsupervised DNA methylation cluster membership was significantly associated with the CF disease status. These observations support a hypothesis that epigenetic changes, specifically DNA methylation in lung macrophages, may participate, at least in part, in driving dysfunctional innate immune cells in the CF lung.

## Methods

### Study population

This study was approved by the Committee for the Protection of Human Subjects at the Geisel School of Medicine at Dartmouth (#22781). All subjects provided written informed consent and were clinically stable and in their baseline state of health. CF subjects had not had a pulmonary exacerbation within the preceding 4 weeks. All subjects were non-smokers.

### Bronchoalveolar lavage and macrophage isolation

Subjects underwent flexible bronchoscopy following local anesthesia with lidocaine to the posterior pharynx and intravenous sedation. A bronchoscope was inserted transorally and advanced through the vocal cords. BAL fluid was obtained from tertiary airways in the right upper and lower lobes (RUL and RLL, respectively). BAL was performed sequentially in the RUL and RLL with 20 ml of sterile saline followed by 10 ml of air, and this was repeated for a total of five times per airway. Lung macrophages were isolated as previously described [41, 42]. Briefly, BAL fluid was filtered through a two-layer gauze, centrifuged, and washed twice in 0.9% NaCl. Cells were counted with a T10-automated cell counter (Bio-Rad, Hercules, CA).

CF BAL cells were incubated with CD15 microbeads and run over an LD column on the QuadroMACS magnet (Miltenyi Biotec, Auburn, CA), according to the manufacturer’s instructions, to deplete neutrophils. Our previous work [41] and the current routine cytospin cell monitoring indicate a final neutrophil population post-negative selection of less than 2% of total BAL cells. Cytospins were performed using a Shandon Cytospin 3 centrifuge (ThermoFisher Scientific, Waltham, MA). Briefly, 75,000 cells resuspended in 200 μl of 0.9% NaCl were loaded into a cytology funnel (Fisher Scientific, Pittsburgh, PA) and centrifuged for 10 min. Cells were allowed to air dry and processed for viewing via Hema 3 Stat pack (Fisher). Imaging was done on an Olympus (Waltham, MA) BX41 microscope with DP2-BSW software (version 2007).

### DNA methylation array

Epigenome-wide DNA methylation profiling was performed via the Infinium Methylation EPIC Bead Chips (Illumina Inc., San Diego, CA) for the determination of methylation levels of more than 850,000 CpG sites as previously described [43]. Briefly, DNA was extracted from bronchoalveolar lavage-derived cells via Qiagen (Germantown, MD) DNeasy Blood and Tissue Kit. DNA was quantitated on a Qubit 3.0 Fluorometer (Life Technologies, Carlsbad, CA). Bisulfite conversion of DNA was carried out with the Zymo EZ DNA methylation kit (Zymo Research, Irvine, CA), and EPIC array hybridization and scanning were performed at the University of Southern California Molecular Genomics Core.

### DNA methylation array data processing

Raw intensity data files (IDATs) from the MethylationEPIC BeadChips were processed by the *minfi* R/Bioconductor analysis pipeline (version 1.21) [44] with annotation file version *ilm10b3.hg19*. Probes associated with known SNPs, non-CpGs and sex chromosomes, as well as those failing to meet a detection *P* value of 0.05 in ≥ 20% samples, were excluded. This pre-processing procedure left 813,096 CpGs with high-quality methylation data in the final data set.

### Targeted, next-generation bisulfite sequencing and data analysis

tNGBS was performed by EpigenDx Inc. (Hopkinton, MA) on the same eight BAL cell specimens as in the EPIC DNA methylation array. Briefly, DNA bisulfite modification was done using EZ-96 DNA methylation kit (Zymo, Irvine, CA) followed by multiplex PCR with Qiagen (Gaithersburg, MD) HotStar Taq and products purified with QIAquick PCR purification kit. Libraries were prepared using the KAPA Library Preparation Kit for Ion Torrent platforms and Ion XpressTM Barcode Adapters (ThermoFisher, Waltham, MA). Library products were purified using Agencourt AMPure XP beads (Beckman Coulter, Indianapolis, IN) and quantified using the Qiagen QIAxcel Advanced System. Barcoded samples were then pooled in an equimolar fashion before template preparation and enrichment were performed on the Ion ChefTM system (ThermoFisher) using Ion 520TM and Ion 530TM Chef reagents. Enriched, template-positive library products were sequenced on the Ion S5TM sequencer using Ion 530TM sequencing chips (ThermoFisher). FASTQ files from the Ion Torrent S5 server were aligned to the local reference database using open-source Bismark Bisulfite Read Mapper with the Bowtie2 alignment algorithm. Methylation levels were calculated in Bismark by dividing the number of methylated reads by the total number of reads.

### Statistical analysis

Unsupervised hierarchical clustering with the Euclidean distance and complete linkage (default) was performed on CpG loci with greatest sample variances. At different variance thresholds, clustering structure appeared to be stable. The recursively partitioned mixture model (RPMM) [45] assuming two terminal clusters was applied to 10,000 most variable CpGs. RPMM was implemented in the *RPMM* R package (version 1.25). A two-sided Fisher’s exact test was used to test the relation of supervised RPMM cluster membership with case-control status. A *P* value of 0.05 was used as the threshold for statistical significance.

The distribution of methylation beta-value variances was examined prior to statistical analysis: 26,733 CpGs with beta-value variance exceeding 0.01 were selected for further investigation. To account for the repeat measurements from a single subject, the correlation coefficient (= 0.762) for the 26,733 CpG beta-values among eight unique subjects (including four cases and four controls) was first calculated by the *duplicateCorrelation* function in the *limma* R/Bioconductor package (v.3.34.9). Differential methylation analysis was carried out by passing logit-transformed beta-values (i.e., *M* values), matched pairs, and associated correlation coefficient into the *lmFit* and *eBayes* functions in *limma*, with adjustment of subject age and sex as fixed effects and subject as a random effect in the model, such that *Y = β*_0_ *+ β*_CF_ *X*_CF_ *+ β*_age_ *X*_age_ *+ β*_sex_ *X*_sex_ *+* RandomEffect(Subject), where *Y* is the methylation beta-values, *β*_0_ is the intercept, *X* is a given covariate, and *β* is the respective model coefficient.

To assess the contribution of cell type heterogeneity to differential methylation, we reconstructed a linear model adjusting for the presence of putative cell types. Briefly, the *RefFreeEWAS* algorithm (R package version 2.1) [16] was applied to 10,000 most variable CpGs across all samples. The proportion of putative cell types were calculated iteratively for the number of such cell types *K* from 2 to 10. The optimal number of putative cell types *K* = 2 was selected as it minimized the variance of the bootstrapped deviance. The difference between cell type proportions were determined by a linear mixed effects model adjusting for age, sex, and repeat measurement, implemented in R packages *lme4* (version 1.1.17) and *lmerTest* (version 2.0.36).

Genomic contexts (Open Sea and Island) were provided in the Illumina EPIC annotation file. The “promoter” transcriptional context was defined as having either a “TSS200” or “TSS1500” annotation, or both in the column *UCSC_RefGene_Group* (TSS, transcription start site). Likewise, the “gene body” transcriptional context was defined as having a “Body” annotation. The “enhancer” context was defined as having a FANTOM4/5 enhancer record or Illumina array enhancer annotation. For each genomic or transcriptional context, odds ratios (OR) for the significant loci relative to the input loci were determined by a two-sided Fisher’s exact test. A *P* value ≤ 0.05 was the threshold for statistical significance. To demonstrate outputs from two different linear models that were similar, a test for enrichment using the Kyoto Encyclopedia of Genes and Genomes (KEGG) was performed using the WebGestalt tool [46]. The differentially methylated CpG loci were used as the input and compared to genes associated with the universe set of CpGs. A pathway was considered significant if the pathway has a Benjamini-Hochberg FDR < 0.05, at least five genes up to a maximum of 2000 genes.

### Code availability

Data processing, statistical analysis, and data visualization R code can be found at github.com/Christensen-Lab-Dartmouth/CF_Epigenetics.

## Additional files

Additional file 1: **Table S1.** Top hypo- and hyper-methylated CpGs in CF that are associated with known genes. (DOCX 17 kb)

Additional file 2: **Figure S1.** Human leukocyte-specific protein-1 (LSP1) gene targeted next-generation sequencing (tNGS) assay region. A tNGS assay was designed for LSP1 surrounding EPIC cg18723409 located in intron 11 of Ensembl Gene ID: ENSG00000130592. A total of 13 CpGs were interrogated in this tNGS assay. (TIFF 638 kb)

Additional file 3: **Table S2.** Targeted next-generation bisulfite sequencing of EPIC identified genes. (DOCX 15 kb)

Additional file 4: **Table S3.** Summary of genomic context related with differentially methylated loci in CF. DHS, DNase I hyper-sensitivity sites. (DOCX 17 kb)

Additional file 5: **Table S4.** Top 10 pathways for 803 unique genes associated with top 5% CpGs based on *P* value in the model without cell type adjustment. (DOCX 16 kb)

Additional file 6: Complete list of DMPs with FDR < 0.05 (CSV 6823 kb)

## Abbreviations

AM: Alveolar macrophage
BAL: Bronchoalveolar lavage
CF: Cystic fibrosis
FDR: False discovery rate
FEV1: Forced expiration volume
KEGG: Kyoto Encyclopedia of Genes and Genomes
RPMM: Recursively partitioned mixture model
tNGS: Targeted next-generation bisulfite sequencing

## References

[1] Bonfield T, Chmiel JF (2017). Impaired innate immune cells in cystic fibrosis: is it really a surprise?. J Cyst Fibros.

[2] Paemka L, McCullagh BN, Abou Alaiwa MH, Stoltz DA, Dong Q, Randak CO (2017). Monocyte derived macrophages from CF pigs exhibit increased inflammatory responses at birth. J Cyst Fibros.

[3] Tarique AA, Sly PD, Holt PG, Bosco A, Ware RS, Logan J (2017). CFTR-dependent defect in alternatively-activated macrophages in cystic fibrosis. J Cyst Fibros.

[4] Simonin-Le Jeune K, Le Jeune A, Jouneau S, Belleguic C, Roux PF, Jaguin M (2013). Impaired functions of macrophage from cystic fibrosis patients: CD11b, TLR-5 decrease and sCD14, inflammatory cytokines increase. PLoS One.

[5] Bruscia EM, Zhang PX, Satoh A, Caputo C, Medzhitov R, Shenoy A (2011). Abnormal trafficking and degradation of TLR4 underlie the elevated inflammatory response in cystic fibrosis. J Immunol.

[6] Kopp BT, Abdulrahman BA, Khweek AA, Kumar SB, Akhter A, Montione R (2012). Exaggerated inflammatory responses mediated by Burkholderia cenocepacia in human macrophages derived from cystic fibrosis patients. Biochem Biophys Res Commun.

[7] Pfeffer KD, Huecksteadt TP, Hoidal JR (1993). Expression and regulation of tumor necrosis factor in macrophages from cystic fibrosis patients. Am J Respir Cell Mol Biol.

[8] Vandivier RW, Fadok VA, Hoffmann PR, Bratton DL, Penvari C, Brown KK (2002). Elastase-mediated phosphatidylserine receptor cleavage impairs apoptotic cell clearance in cystic fibrosis and bronchiectasis. J Clin Invest.

[9] Vandivier RW, Fadok VA, Ogden CA, Hoffmann PR, Brain JD, Accurso FJ (2002). Impaired clearance of apoptotic cells from cystic fibrosis airways. Chest.

[10] Bonfield TL, Panuska JR, Konstan MW, Hilliard KA, Hilliard JB, Ghnaim H (1995). Inflammatory cytokines in cystic fibrosis lungs. Am J Respir Crit Care Med.

[11] Osika E, Cavaillon JM, Chadelat K, Boule M, Fitting C, Tournier G (1999). Distinct sputum cytokine profiles in cystic fibrosis and other chronic inflammatory airway disease. Eur Respir J.

[12] Sagel SD, Chmiel JF, Konstan MW (2007). Sputum biomarkers of inflammation in cystic fibrosis lung disease. Proc Am Thorac Soc.

[13] Wu C, Morris JR (2001). Genes, genetics, and epigenetics: a correspondence. Science.

[14] Marsit CJ, Brummel SS, Kacanek D, Seage GR, Spector SA, Armstrong DA (2015). Infant peripheral blood repetitive element hypomethylation associated with antiretroviral therapy in utero. Epigenetics.

[15] Morandini AC, Santos CF, Yilmaz O. Role of epigenetics in modulation of immune response at the junction of host-pathogen interaction and danger molecule signaling. Pathog Dis. 2016;74(7):ftw082. 10.1093/femspd/ftw082 PMC5985482 27542389

[16] Houseman EA, Kile ML, Christiani DC, Ince TA, Kelsey KT, Marsit CJ (2016). Reference-free deconvolution of DNA methylation data and mediation by cell composition effects. BMC Bioinformatics.

[17] Dewhurst JA, Lea S, Hardaker E, Dungwa JV, Ravi AK, Singh D (2017). Characterisation of lung macrophage subpopulations in COPD patients and controls. Sci Rep.

[18] Magalhaes M, Rivals I, Claustres M, Varilh J, Thomasset M, Bergougnoux A (2017). DNA methylation at modifier genes of lung disease severity is altered in cystic fibrosis. Clin Epigenetics.

[19] Logie C, Stunnenberg HG (2016). Epigenetic memory: a macrophage perspective. Semin Immunol.

[20] Cabanel M, Brand C, Oliveira-Nunes MC, Cabral-Piccin MP, Lopes MF, Brito JM (2015). Epigenetic control of macrophage shape transition towards an atypical elongated phenotype by histone deacetylase activity. PLoS One.

[21] Kittan NA, Allen RM, Dhaliwal A, Cavassani KA, Schaller M, Gallagher KA (2013). Cytokine induced phenotypic and epigenetic signatures are key to establishing specific macrophage phenotypes. PLoS One.

[22] Gharib SA, Vaisar T, Aitken ML, Park DR, Heinecke JW, Fu X (2009). Mapping the lung proteome in cystic fibrosis. J Proteome Res.

[23] Simhadri VL, Hansen HP, Simhadri VR, Reiners KS, Bessler M, Engert A (2012). A novel role for reciprocal CD30-CD30L signaling in the cross-talk between natural killer and dendritic cells. Biol Chem.

[24] Nicod LP, Isler P (1997). Alveolar macrophages in sarcoidosis coexpress high levels of CD86 (B7.2), CD40, and CD30L. Am J Respir Cell Mol Biol.

[25] Kim HJ, Park J, Lee SK, Kim KR, Park KK, Chung WY (2015). Loss of RUNX3 expression promotes cancer-associated bone destruction by regulating CCL5, CCL19 and CXCL11 in non-small cell lung cancer. J Pathol.

[26] Aldinucci D, Colombatti A (2014). The inflammatory chemokine CCL5 and cancer progression. Mediat Inflamm.

[27] Donato R (2003). Intracellular and extracellular roles of S100 proteins. Microsc Res Tech.

[28] Xia C, Braunstein Z, Toomey AC, Zhong J, Rao X (2017). S100 proteins as an important regulator of macrophage inflammation. Front Immunol.

[29] Maxeiner S, Shi N, Schalla C, Aydin G, Hoss M, Vogel S (2015). Crucial role for the LSP1-myosin1e bimolecular complex in the regulation of Fcgamma receptor-driven phagocytosis. Mol Biol Cell.

[30] Barrow AD, Palarasah Y, Bugatti M, Holehouse AS, Byers DE, Holtzman MJ (2015). OSCAR is a receptor for surfactant protein D that activates TNF-alpha release from human CCR2+ inflammatory monocytes. J Immunol.

[31] Merck E, Gaillard C, Scuiller M, Scapini P, Cassatella MA, Trinchieri G (2006). Ligation of the FcR gamma chain-associated human osteoclast-associated receptor enhances the proinflammatory responses of human monocytes and neutrophils. J Immunol.

[32] Auffray C, Sieweke MH, Geissmann F (2009). Blood monocytes: development, heterogeneity, and relationship with dendritic cells. Annu Rev Immunol.

[33] Gardiner-Garden M, Frommer M (1987). CpG islands in vertebrate genomes. J Mol Biol.

[34] Pennacchio LA, Bickmore W, Dean A, Nobrega MA, Bejerano G (2013). Enhancers: five essential questions. Nat Rev Genet.

[35] Mohrs M, Blankespoor CM, Wang ZE, Loots GG, Afzal V, Hadeiba H (2001). Deletion of a coordinate regulator of type 2 cytokine expression in mice. Nat Immunol.

[36] Kolahian S, Oz HH, Zhou B, Griessinger CM, Rieber N, Hartl D (2016). The emerging role of myeloid-derived suppressor cells in lung diseases. Eur Respir J.

[37] Rieber N, Brand A, Hector A, Graepler-Mainka U, Ost M, Schafer I (2013). Flagellin induces myeloid-derived suppressor cells: implications for Pseudomonas aeruginosa infection in cystic fibrosis lung disease. J Immunol.

[38] Ost M, Singh A, Peschel A, Mehling R, Rieber N, Hartl D (2016). Myeloid-derived suppressor cells in bacterial infections. Front Cell Infect Microbiol.

[39] Oz HH, Zhou B, Voss P, Carevic M, Schroth C, Frey N (2016). Pseudomonas aeruginosa airway infection recruits and modulates neutrophilic myeloid-derived suppressor cells. Front Cell Infect Microbiol.

[40] Zheng SC, Beck S, Jaffe AE, Koestler DC, Hansen KD, Houseman AE (2017). Correcting for cell-type heterogeneity in epigenome-wide association studies: revisiting previous analyses. Nat Methods.

[41] Bessich JL, Nymon AB, Moulton LA, Dorman D, Ashare A (2013). Low levels of insulin-like growth factor-1 contribute to alveolar macrophage dysfunction in cystic fibrosis. J Immunol.

[42] Monick MM, Powers LS, Barrett CW, Hinde S, Ashare A, Groskreutz DJ (2008). Constitutive ERK MAPK activity regulates macrophage ATP production and mitochondrial integrity. J Immunol.

[43] Kling T, Wenger A, Beck S, Caren H (2017). Validation of the MethylationEPIC BeadChip for fresh-frozen and formalin-fixed paraffin-embedded tumours. Clin Epigenetics.

[44] Aryee MJ, Jaffe AE, Corrada-Bravo H, Ladd-Acosta C, Feinberg AP, Hansen KD (2014). Minfi: a flexible and comprehensive Bioconductor package for the analysis of Infinium DNA methylation microarrays. Bioinformatics.

[45] Houseman EA, Christensen BC, Yeh RF, Marsit CJ, Karagas MR, Wrensch M (2008). Model-based clustering of DNA methylation array data: a recursive-partitioning algorithm for high-dimensional data arising as a mixture of beta distributions. BMC Bioinformatics.

[46] Wang J, Vasaikar S, Shi Z, Greer M, Zhang B (2017). WebGestalt 2017: a more comprehensive, powerful, flexible and interactive gene set enrichment analysis toolkit. Nucleic Acids Res.

