# Supplementary material for: Genome-wide DNA methylation profiling shows a distinct epigenetic signature associated with lung macrophages in cystic fibrosis
Source: Clin Epigenetics. 2018 Dec 10;10:152. doi: 10.1186/s13148-018-0580-2 (PMC6288922; doi:10.1186/s13148-018-0580-2)
Supplement: Supplementary file 1 — Table S1. Top hypo- and hyper-methylated CpGs in CF that are associated with known genes. (DOCX 17 kb) [file 13148_2018_580_MOESM1_ESM.docx]

Table S1. Top Hypo- and Hyper-methylated CpGs in CF that are associated with known genes.

| **Name** | **Chromosome** | **Coordinate** | **Strand** | **Genomic Context** | **Gene** | **∆beta-value** | **Log FC** | **P-value** | **FDR P-value** |
| --- | --- | --- | --- | --- | --- | --- | --- | --- | --- |
| cg03132824 | chr4 | 3373006 | - | N_Shore | *RGS12* | 0.40 | 4.51 | 1.63E-04 | 0.018 |
| cg10912203 | chr9 | 117656633 | + | OpenSea | *TNFSF8* | 0.37 | 4.29 | 1.23E-04 | 0.018 |
| cg26972832 | chr6 | 24437460 | - | OpenSea | *GPLD1* | 0.25 | 4.07 | 7.19E-04 | 0.018 |
| cg14168080 | chr7 | 157504135 | + | Island | *PTPRN2* | 0.24 | 3.83 | 9.85E-04 | 0.019 |
| cg10317717 | chr7 | 48129904 | + | Island | *UPP1* | 0.24 | 3.82 | 1.29E-02 | 0.043 |
| cg08477332 | chr1 | 153590243 | - | OpenSea | *S100A14* | -0.31 | -4.93 | 2.30E-08 | 0.001 |
| cg26427109 | chr11 | 60739019 | - | OpenSea | *CD6* | -0.42 | -4.91 | 2.19E-04 | 0.018 |
| cg11738485 | chr19 | 12877000 | + | Island | *HOOK2* | -0.32 | -4.88 | 4.01E-02 | 0.078 |
| cg06888746 | chr10 | 105517764 | + | OpenSea | *SH3PXD2A* | -0.28 | -4.58 | 1.38E-03 | 0.020 |
| cg18723409 | chr11 | 1911547 | - | OpenSea | *LSP1* | -0.44 | -4.58 | 1.48E-04 | 0.018 |

∆beta, % methylated allele in Healthy control - CF. FDR, false discovery rate.
