# Supplementary figures and images for: Genome-wide DNA methylation profiling shows a distinct epigenetic signature associated with lung macrophages in cystic fibrosis
Source: Clin Epigenetics. 2018 Dec 10;10:152. doi: 10.1186/s13148-018-0580-2 (PMC6288922; doi:10.1186/s13148-018-0580-2)

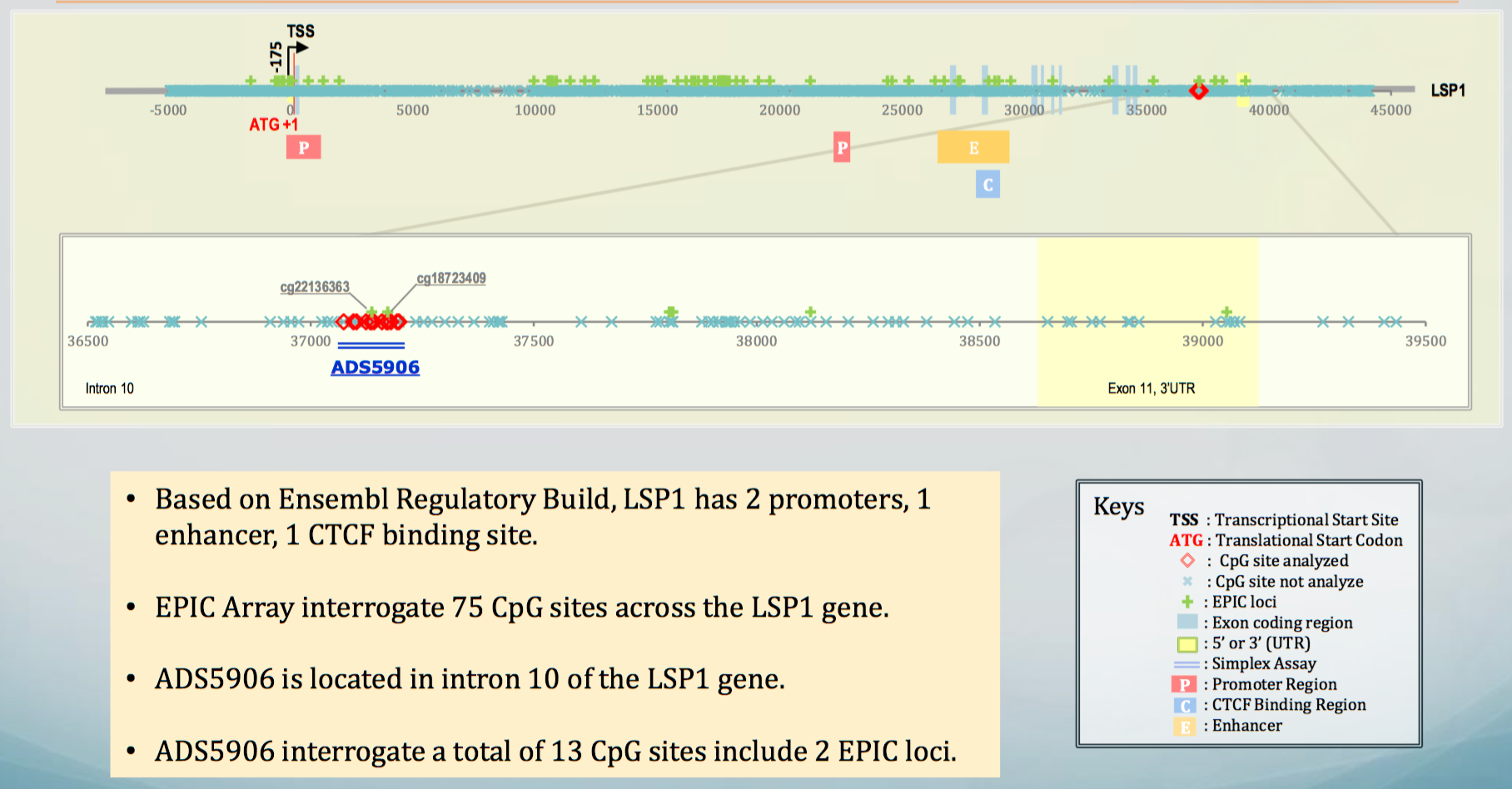

Supplement: Supplementary file 2 — Figure S1. Human leukocyte-specific protein-1 (LSP1) gene targeted next-generation sequencing (tNGS) assay region. A tNGS assay was designed for LSP1 surrounding EPIC cg18723409 located in intron 11 of Ensembl Gene ID: ENSG00000130592. A total of 13 CpGs were interrogated in this tNGS assay. (TIFF 638 kb) [file 13148_2018_580_MOESM2_ESM.tiff]
