# Supplementary material for: Genome-wide DNA methylation profiling shows a distinct epigenetic signature associated with lung macrophages in cystic fibrosis
Source: Clin Epigenetics. 2018 Dec 10;10:152. doi: 10.1186/s13148-018-0580-2 (PMC6288922; doi:10.1186/s13148-018-0580-2)
Supplement: Supplementary file 3 — Table S2. Targeted next-generation bisulfite sequencing of EPIC identified genes. (DOCX 15 kb) [file 13148_2018_580_MOESM3_ESM.docx]

Table S2. Targeted next generation bisulfite sequencing of EPIC identified genes.

____________________________________________________________________________________________________

**EPIC CpG with tNGS All CpGs in tNGS Assay**

**Avg. Samp. % Meth. % Meth. Δbeta % Meth. % Meth. Δbeta**

**Gene Location # CpG Sites Reads Healthy CF value Healthy CF value**

CD6 Chr11: 60971523-60971547 3 1755 78.2 43.8 (-) 0.344 74.6 39.7 (-) 0.349

HOOK2 Chr19: 12766206-12766077 16 1906 56.6 24.6 (-) 0.32 57.2 25.5 (-) 0.317

LSP1 Chr11: 1890218-1890342 13 468 73.0 43.8 (-) 0.292 59.5 32.0 (-) 0.275

RGS12 Chr4: 337121-3371370 9 649 13.0 42.2 (+) 0.291 13.4 44.7 (+) 0.313

SH3PXD2A Chr10: 103758048-1037579587 7 646 22.2 3.8 (-) 0.184 32.7 8.2 (-) 0.245

UPP1 Chr7: 1890218-1890342 12 423 2.5 28.8 (+) 0.263 3.6 29.6 (+) 0.26

____________________________________________________________________________________________________
