# Supplementary material for: Genome-wide DNA methylation profiling shows a distinct epigenetic signature associated with lung macrophages in cystic fibrosis
Source: Clin Epigenetics. 2018 Dec 10;10:152. doi: 10.1186/s13148-018-0580-2 (PMC6288922; doi:10.1186/s13148-018-0580-2)
Supplement: Supplementary file 4 — Table S3. Summary of genomic context related with differentially methylated loci in CF. DHS, DNase I hyper-sensitivity sites. (DOCX 17 kb) [file 13148_2018_580_MOESM4_ESM.docx]

Table S3. Summary of genomic context related with differentially methylated loci

in CF. DHS, DNase I hypersensitivity sites.

|  | HYPOmethylated (58 loci) | HYPERmethylated (51 loci) | Input set  (26,733 loci) |
| --- | --- | --- | --- |
| Promoter | 10 (17.2%) | 2 (3.92%) | 3715 (13.9%) |
| Enhancer | 24 (41.4%) | 11 (21.6%) | 7572 (28.3%) |
| DHS | 44 (75.9%) | 42 (82.4%) | 18803 (70.3%) |
| Island | 10 (17.2%) | 4 (7.8%) | 1824 (6.8%) |
| OpenSea | 40 (69.0%) | 42 (82.4%) | 19151 (71.6%) |
