# Supplementary material for: Genome-wide DNA methylation profiling shows a distinct epigenetic signature associated with lung macrophages in cystic fibrosis
Source: Clin Epigenetics. 2018 Dec 10;10:152. doi: 10.1186/s13148-018-0580-2 (PMC6288922; doi:10.1186/s13148-018-0580-2)
Supplement: Supplementary file 5 — Table S4. Top 10 pathways for 803 unique genes associated with top 5% CpGs based on P value in the model without cell type adjustment. (DOCX 16 kb) [file 13148_2018_580_MOESM5_ESM.docx]

Table S4. Top 10 pathways for 803 unique genes associated with top 5% CpGs based on *P*-value

In the model without cell type adjustment

__________________________________________________________________________________________

KEGG ID Description Total Expected Observed *P*-value FDR

# genes ratio ratio

__________________________________________________________________________________________

hsa01040 Biosynthesis of unsaturated fatty acids 6 1.25 4.81 5.72E-04 0.14

hsa00561 Glycerolipid metabolism 9 2.84 3.17 1.08E-03 0.14

hsa04666 Fc gamma R-mediated phagocytosis 15 6.58 2.28 1.47E-03 0.14

hsa04664 Fc epsilon RI signaling pathway 10 4.54 2.20 1.15E-02 0.57

hsa05220 Chronic myeloid leukemia 10 4.54 2.20 1.15E-02 0.57

hsa05222 Small cell lung cancer 11 5.22 2.11 1.16E-02 0.57

hsa05206 MicroRNAs in cancer 18 10.66 1.69 1.63E-02 0.66

hsa00062 Fatty acid elongation 4 1.13 3.53 1.96E-02 0.66

hsa04961 Endocrine and other factor-regulated 7 2.95 2.37 2.21E-02 0.66

calcium reabsorption

hsa00564 Glycerolphospholipid metabolism 10 4.99 2.00 2.23E-02 0.66

__________________________________________________________________________________________
